# Genetic basis of rifampicin resistance in methicillin-resistant *Staphylococcus aureus *suggests clonal expansion in hospitals in Cape Town, South Africa

**DOI:** 10.1186/1471-2180-12-46

**Published:** 2012-03-26

**Authors:** Melissa J Jansen van Rensburg, Andrew C Whitelaw, Brenda G Elisha

**Affiliations:** 1 Division of Medical Microbiology, Faculty of Health Sciences, University of Cape Town, Anzio Road, Observatory, Cape Town 7925, South Africa; 2 National Health Laboratory Service, Groote Schuur Hospital, Anzio Road, Observatory, Cape Town 7925, South Africa; 3 National Institute for Communicable Diseases, National Health Laboratory Service, University of Cape Town, Anzio Road, Observatory, Cape Town 7925, South Africa; 4 Current address: Department of Zoology, University of Oxford, The Tinbergen Building, South Parks Road, Oxford OX1 3PS, UK

## Abstract

**Background:**

Since 2001, several studies have reported high rifampicin resistance rates (45 - 100%) among methicillin-resistant *Staphylococcus aureus *(MRSA) isolates from South Africa. The authors previously characterised 100 MRSA isolates from hospitals in Cape Town, South Africa; forty-five percent of these isolates were rifampicin-resistant. The majority (44/45) corresponded to ST612-MRSA-IV, which is prevalent in South Africa, but has not been reported frequently elsewhere. The remaining rifampicin-resistant isolate corresponded to ST5-MRSA-I. The aim of this study was to investigate further the prevalence and genetic basis of rifampicin-resistance in MRSA isolates from hospitals in Cape Town.

**Results:**

Between July 2007 and June 2011, the prevalence of rifampicin-resistant MRSA in hospitals in Cape Town ranged from 39.7% to 46.4%. Based on the results of the aforementioned study, nine ST612-MRSA-IV isolates, the rifampicin-resistant ST5-MRSA-I isolate, and two rifampicin-susceptible MRSA isolates were investigated. Four previously described ST612-MRSA-IV isolates, including two each from South Africa and Australia, were also included.

The ST5-MRSA-I isolate carried a single mutational change, H_481_Y, commonly associated with high-level rifampicin resistance. All ST612-MRSA-IV isolates carried an uncommon double amino acid substitution in RpoB, H_481_N, I_527_M, whilst one of the Australian ST612-MRSA-IV isolates carried an additional mutation within *rpoB*, representing a novel *rpoB *genotype: H_481_N, I_527_M, K_579_R. All ST612-MRSA-IV isolates also shared a unique silent single nucleotide polymorphism (SNP) within *rpoB*.

**Conclusions:**

That local ST612-MRSA-IV isolates described here share an uncommon *rpoB *genotype and a unique silent SNP suggests this clone may have undergone clonal expansion in hospitals in Cape Town. Further, the data suggest that these isolates may be related to rifampicin-resistant ST612-MRSA-IV previously described in South Africa and Australia.

## Background

It has long been acknowledged that antimicrobial use drives the emergence of resistant pathogens [1]. Currently in South Africa, rifampicin is used primarily for the treatment of tuberculosis, although it is also sometimes used in combination therapies to treat *Staphylococcus aureus *infections. A national antimicrobial susceptibility surveillance study carried out in South Africa between 2005 and 2006 showed that 52.8% of MRSA isolates from public diagnostic laboratories were rifampicin-resistant [2]. Regional studies carried out between 2001 and 2006 in public hospitals in the Kwa-Zulu Natal and Gauteng provinces of South Africa reported that 63 - 100% of MRSA isolates were rifampicin-resistant [3,4]. Given South Africa's high incidence of tuberculosis and subsequent widespread use of rifampicin, it is likely that selective pressure has propelled the emergence and preponderance of rifampicin-resistant MRSA in this country.

A recent study on the molecular characterisation of MRSA from hospitals in Cape Town, South Africa, showed that ST612-MRSA-IV, a previously infrequently reported clone, was dominant in Cape Town hospitals [5]. Of the 100 MRSA isolates included in that study, 45 were rifampicin-resistant. Moreover, ST612-MRSA-IVaccounted for 44 of these rifampicin-resistant isolates. The remaining rifampicin-resistant MRSA isolate corresponded to ST5-MRSA-I. A recent national report on MRSA clones circulating in South Africa indicated that ST612-MRSA-IV was the most prevalent and widespread clone [6]. However, whether these MRSA isolates were resistant to rifampicin was not reported. Prior to the Cape Town study [5] and the recently reported national investigation [6], only four clinical ST612-MRSA-IV isolates had been described, including two each from South Africa and Australia, although the antimicrobial susceptibility profiles of these isolates were not reported [7-9].

Rifampicin is a bactericidal antimicrobial agent that inhibits transcription by binding to the β-subunit of the bacterial DNA-dependent RNA polymerase [10]. The β-subunit of RNA polymerase is encoded by *rpoB*, and mutations within conserved regions of the gene have been shown to confer resistance to rifampicin in a number of bacteria, including *S. aureus *[10-12]. The majority of mutations associated with rifampicin resistance in *S. aureus *have been mapped to a conserved region of *rpoB *known as the rifampicin resistance-determining region (RRDR) [11-13].

The available information on rifampicin resistance genotypes in *S. aureus *is restricted to a limited number of studies [11-17], which, to the best of our knowledge, have included only one isolate from South Africa [17]. This communication describes the prevalence and genetic basis of rifampicin resistance in MRSA from hospitals in Cape Town.

## Methods

### Setting and statistical analysis of laboratory data

The National Health Laboratory Service (NHLS) microbiology laboratory at Groote Schuur Hospital, Cape Town, serves three tertiary- and two secondary-level public hospitals situated within Cape Town. The laboratory data for all *S. aureus *isolates collected between July 2007 and June 2011 were retrieved from the NHLS database. The isolates were stratified according to methicillin and rifampicin susceptibilities. Differences between proportions were analysed using the *χ*^2^-test, and the *χ*^2^-test for trend was used to assess linear trends over time [18].

### Isolate selection

*S. aureus *isolates were identified either by the production of DNAse, or on the VITEK 2 (bioMérieux, La Balme-les-Grottes, France). The authors recently used a combination of antimicrobial susceptibility testing, pulsed-field gel electrophoresis (PFGE), SCC*mec *typing, *spa *typing and multilocus sequence typing (MLST) to characterise 100 MRSA isolates obtained from hospitals in Cape Town between January 2007 and December 2008 [5]. The majority of the isolates were obtained from two tertiary level facilities, Groote Schuur Hospital (GSH) and Red Cross War Memorial Children's Hospital (RCCH). Forty-five of the 100 isolates were rifampicin-resistant (44 ST612-MRSA-IV and 1 ST5-MRSA-I) [5].

Twelve of the previously characterised MRSA isolates described above were selected for *rpoB *genotyping, and their properties are shown in Table 1. Two ST612-MRSA-IV isolates, one each from GSH and RCCH, were selected from PFGE cluster D [5]. Both had *spa *type t064, the only type detected in representative isolates from this cluster [5]. Five ST612 MRSA-IV isolates, from four of the five hospitals (Table 1), were selected from the more genetically diverse PFGE cluster E [5]. Three *spa *types were identified in representative isolates from cluster E, with t1443 most frequently detected. Two of four sporadic ST612-MRSA-IV isolates were included. These isolates were obtained from GSH and RCCH, with one corresponding to *spa *type t1257, which was not identified in any of the other ST612-MRSA-IV isolates (Table 1) [5]. Also included were the rifampicin-resistant ST5-MRSA-I and two rifampicin-susceptible isolates (Table 1). Additionally, two ST612-MRSA-IV from both South Africa (N83 and N84) [8] and Australia (04-17052 and 09-15534) [9] were included in the investigations (Table 1).

**Table 1:** Characteristics of MRSA isolates selected for *rpoB *genotyping

Clonal type^1 ^(isolate name)	PFGE cluster^2 ^(n)	*spa *type	Rifampicin phenotype^3 ^	Geographical origin	Hospital^4 ^(n)/Year of isolation	Reference
ST612-MRSA-IV	D (2)	t064	Resistant	Cape Town, RSA^5^	GSH (1), RCCH (1)/2008	[5]

ST612-MRSA-IV	E (1)	t064	Resistant	Cape Town, RSA	UCTPH/2008	[5]

ST612-MRSA-IV	E (4)	t1443	Resistant	Cape Town, RSA	GSH (2), RCCH (1), VH (1)/2008	[5]

ST612-MRSA-IV	Sporadic isolate (1)	t1443	Resistant	Cape Town, RSA	GSH/2008	[5]

ST612-MRSA-IV	Sporadic isolate (1)	t1257	Resistant	Cape Town, RSA	RCCH/2008	[5]

ST5-MRSA-I	C (1)	t045	Resistant	Cape Town, RSA	MMH/2008	[5]

ST22-MRSA-IV	Sporadic isolate (1)	t032	Susceptible	Cape Town, RSA	GSH/2008	[5]

ST36-MRSA-II	F (1)	t021	Susceptible	Cape Town, RSA	GSH/2007	[5]

ST612-MRSA-IV (N83, N84)	ND^6 ^(2)	t064	Resistant	RSA	Unknown/2004 - 2005	[8]

ST612-MRSA-IV (04-17052)	ND (1)	t064	Resistant	Perth, Australia	Unknown/2004	[9]

ST612-MRSA-IV (09-15534)	ND (1)	t7571	Resistant	Perth, Australia	Unknown/2009	[9]

^1 ^Clonal types are indicated using the current international nomenclature (sequence type (ST) -antimicrobial phenotype - staphylococcal cassette chromosome *mec *(SCC*mec*) type)

^2 ^PFGE, pulsed-field gel electrophoresis

^3 ^As determined by disc diffusion or on the VITEK 2

^4 ^GSH, Groote Schuur Hospital; RCCH, Red Cross War Memorial Children's Hospital; UCTPH, University of Cape Town Private Academic Hospital; VH, Victoria Hospital; MMH, Mowbray Maternity Hospital

^5 ^RSA, Republic of South Africa

^6 ^ND, not determined

### Antimicrobial susceptibility testing

The rifampicin and vancomycin MICs of the study isolates were determined by E-test (bioMérieux, La Balme-les-Grottes, France).

### *rpoB *genotyping

A 702 bp region of *rpoB *spanning amino acid residues 441 to 673 (*S. aureus *co-ordinates), including the RRDR, was amplified by PCR using primers designed by Aubry-Damon *et al. *[11]. The PCR was carried out in a final volume of 100 μl with 1X reaction buffer, 1.5 mM MgCl_2_, 40 pmol of each primer and 400 μM of deoxynucleotide triphosphates (Thermo Scientific, Wilmington, DE, USA). One hundred nanograms of template DNA and 1 U of Super Therm *Taq *DNA polymerase (JMR Holdings, London, UK) were added to each reaction. Amplification was carried out using an Applied Biosystems 2720 Thermocycler (Applied Biosystems, Carlsbad, CA, USA). The PCR cycling conditions consisted of an initial denaturation step at 94°C for 4 min, followed by 35 cycles of denaturation at 94°C for 30 s, annealing at 52°C for 45 s, elongation at 72°C for 45 s, with a final extension step at 72°C for 3 min.

The 702 bp fragment was purified using the MinElute Gel Extraction Kit (QIAGEN, Valencia, CA, USA), and both strands were sequenced directly at the Central Analytical Facility at the University of Stellenbosch. The nucleotide sequences obtained were aligned to the *rpoB *sequence of rifampicin-susceptible *S. aureus *strain RN4220 (GenBank accession number: X64172) using the ClustalW algorithm in BioEdit Sequence Alignment Editor (version 7.0.5.2) [19].

## Results

### Antimicrobial susceptibility testing

The 14 rifampicin-resistant isolates expressed high-level rifampicin resistance (rifampicin MICs ≥ 256 mg/L). The remaining 2 isolates were confirmed to be rifampicin-susceptible by E-test (rifampicin MICs ≤ 0.016 mg/L), as was previously determined by disc diffusion or on the VITEK 2 [5]. All 16 isolates were susceptible to vancomycin; 15 had vancomycin MICs ≤ 1 mg/L and one isolate, CT-C31-08 (ST5-MRSA-I), had a vancomycin MIC of 2 mg/L.

### Prevalence of rifampicin resistance among *S. aureus *isolates from hospitals in Cape Town

**Figure 1:**
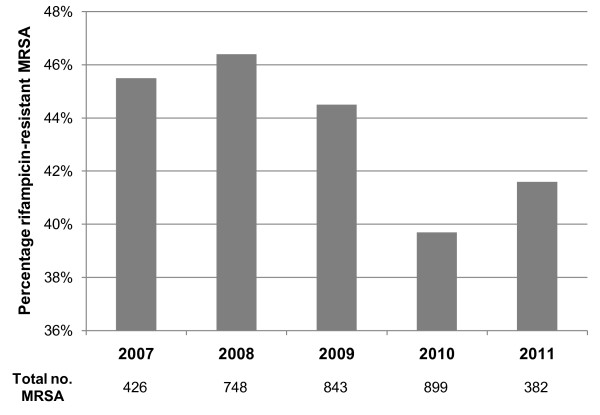
**Annual percentage of rifampicin-resistant MRSA isolates collected between July 2007 and June 2011**. Figures shown below the graph indicate the total number of MRSA isolates obtained each year, or part thereof. No significant difference was detected in the prevalence of rifampicin resistance among MRSA isolates over the four year period (*p *= 0.0521).

The NHLS microbiology laboratory at Groote Schuur Hospital carried out antimicrobial susceptibility testing on 13 746 clinical *S. aureus *isolates between July 2007 and June 2011. MRSA accounted for 3298 (24%) of all *S. aureus *isolates. Overall, 328 (3.1%) of the methicillin-susceptible *S. aureus *(MSSA) isolates were resistant to rifampicin, while 1432 (43.4%) of the MRSA isolates were rifampicin-resistant (*p *< 0.0001). No significant difference was detected in the prevalence of rifampicin resistance among MRSA isolates over the four year period (*p *= 0.0521), as illustrated in Figure 1.

### Identification of mutations in *rpoB*

The *rpoB *genotypes (GenBank accession numbers JN593081 - JN593085) and other molecular characteristics of the 16 isolates included in this investigation are shown in Table 2. No amino acid substitutions were observed in the RpoB protein sequences of the rifampicin-susceptible isolates. The ST5-MRSA-I isolate carried a single H_481_Y substitution known to confer high-level rifampicin resistance [11,12] (Table 2). The nine ST612-MRSA-IV isolates from hospitals in Cape Town all carried the same double mutational changes within the RRDR, H_481_N, I_527_M, which have also previously been associated with high-level rifampicin resistance in *S. aureus *[12,17]. N83 and N84, the ST612-MRSA-IV isolates previously identified in South Africa, also carried these changes. Similarly, the H_481_N, I_527_M double substitution was observed in 04-17052 and 09-15534, the two ST612-MRSA-IV isolates from Australia; however, an additional novel amino acid substitution, K_579_R, was observed outside the RRDR in isolate 09-15534 (Table 2).

**Table 2:** Results of rifampicin susceptibility testing and *rpoB *genotyping

Clonal type^1 ^(clonal complex)	PFGE cluster^2 ^(n)/*spa *type (n)	Isolate origin (isolate name)	Rifampicin MIC (mg/L)^3^	Amino acid position^4^	Nucleotide substitution	Amino acid substitution
ST22- MRSA-IV (22)	Sporadic isolate (1)/t032 (1)	Cape Town, RSA^5^	≤ 0.016	498	GCG → GCT	-
				554	CAT → CAC	-
				599	AAT → AAC	-

ST36- MRSA-II (30)	F (1)/t021 (1)	Cape Town, RSA	≤ 0.016	474	AAC → AAT	-
				498	GCG → GCT	-
				502	GTA → GTG	-
				518	ACA → ACG	-

ST5- MRSA-I (5)	C (1)/t045 (1)	Cape Town, RSA	≥ 256	481	CAT → TAT	H_481_Y
				498	GCG → GCT	-
				630	AAT → AAC	-
				658	GGT → GGA	-

ST612- MRSA-IV (8)	D (2), E (5), sporadic isolates (2)/t064 (3), t1443 (5), t1257 (1)	Cape Town, RSA	≥ 256	481	CAT → AAT	H_481_N
				498	GCG → GCT	-
				512	CGT → CGC	-
				527	ATT → ATG	I_527_M

ST612- MRSA-IV (8)	ND^6 ^(2)/t064 (2)	RSA (N83; N84)	≥ 256	481	CAT → AAT	H_481_N
				498	GCG → GCT	-
				512	CGT → CGC	-
				527	ATT → ATG	I_527_M

ST612- MRSA-IV (8)	ND (1)/t064 (1)	Australia (04-17052)	≥ 256	481	CAT → AAT	H_481_N
				498	GCG → GCT	-
				512	CGT → CGC	-
				527	ATT → ATG	I_527_M

ST612- MRSA-IV (8)	ND (1)/t7571 (1)	Australia (09-15534)	≥ 256	481	CAT → AAT	H_481_N
				498	GCG → GCT	-
				512	CGT → CGC	-
				527	ATT→ATG	I_527_M
				579	AAA→AGA	K_579_R

^1 ^Clonal types are indicated using the current international nomenclature (sequence type (ST) - antimicrobial phenotype - staphylococcal cassette chromosome *mec *(SCC*mec*) type)

^2 ^PFGE, pulsed-field gel electrophoresis

^3 ^As determined by E-test

^4 ^*S. aureus *co-ordinates

^5 ^RSA, Republic of South Africa

^6 ^ND, not determined

In addition to the mutations associated with amino acid substitutions in RpoB, silent single nucleotide polymorphisms (SNPs) were detected in the *rpoB *sequences of all 16 isolates (Table 2). Based on a comparison with the corresponding sequence of the rifampicin-susceptible *S. aureus *strain RN4220, all isolates shared a common SNP at amino acid 498 (GCG → GCT), as shown in Table 2. Otherwise between one and three additional SNPs particular to each clonal type were identified. Of note is the conserved SNP at amino acid 512 (CGT → CGC), which was detected in all 13 ST612-MRSA-IV isolates (Table 2).

## Discussion

A number of factors drive the emergence and spread of antibiotic resistance, including antibiotic usage, infection control practices and the organism's genetics [1]. Previous studies carried out in South Africa have reported large proportions of rifampicin-resistant MRSA isolates [2-5], and this study is no exception with the prevalence of rifampicin-resistance among MRSA isolates ranging from 39.7% to 46.4% (Figure 1). It is likely that the frequent use of rifampicin to treat tuberculosis in South Africa has driven the high prevalence of rifampicin-resistance among local MRSA. Support for this suggestion comes from the work of Sekiguchi *et al. *[14] who reported a significantly higher prevalence of rifampicin-resistant MRSA in tuberculosis wards compared to non-tuberculosis wards in two hospitals in Japan.

A previous study showed that ST612-MRSA-IV was the dominant clone circulating in public hospitals in Cape Town. The 44 isolates corresponding to this clonal type were uniformly resistant to rifampicin. Only one other isolate of the 100 MRSA investigated was resistant to this antibiotic and corresponded to ST5-MRSA-I [5]. Analysis of the RRDR of 14 rifampicin-resistant MRSA (rifampicin MICs ≥ 256 mg/L), including the ST5-MRSA-I isolate, nine representatives of Cape Town ST612-MRSA-IV isolates and four previously described ST612-MRSA-IV isolates, identified three *rpoB *genotypes; no amino acid substitutions were detected in the two rifampicin-susceptible isolates (rifampicin MICs ≤ 0.016 mg/L) (Table 2).

The high-level rifampicin-resistant ST5-MRSA-I isolate carried a single mutational change within RpoB, H_481_Y. This substitution, previously associated with high-level resistance, is one of the most common rifampicin resistance genotypes and has been reported previously in several laboratory mutants and clinical isolates [11-13,16,17]. Molecular modelling has demonstrated that the H_481_Y substitution disrupts an H bond between rifampicin and RNA polymerase, and also reduces hydrophobic interactions within the binding cavity, thereby decreasing the affinity of the drug for its target [13].

A relatively uncommon genotype, H_481_N, I_527_M, previously reported in two clinical rifampicin-resistant MRSA from Italy [12] and a single vancomycin intermediate *S. aureus *(VISA) isolate from Brazil [17], accounted for 12 of the 13 high-level rifampicin-resistant ST612-MRSA-IV isolates, including N83, N84 and 04-17052. These results differ from the findings of Mick *et al. *[15] who detected four markedly different rifampicin resistance genotypes among 32 ST228-MRSA-IV isolates, expressing various levels of resistance, which were collected from a single hospital over three years.

The third *rpoB *genotype, H_481_N, I_527_M, K_579_R, was present in 09-15534, the remaining Australian ST612-MRSA-IV isolate. To the best of our knowledge, K_579_R, which occurs outside the RRDR, has not been reported previously, hence H_481_N, I_527_M, K_579_R represents a novel *rpoB *genotype. Whether the latter substitution impacts rifampicin resistance is unknown because the RRDR of this isolate contains two other mutations associated with resistance to this antibiotic. It is possible that this novel K_579_R substitution represents the latest mutational change in ST612-MRSA-IV as isolate 09-15534 was isolated in 2009, whereas the other MRSA strains included in this study were collected between 2004 and 2008.

A number of silent SNPs were detected in the 16 isolates when using the nucleotide sequence of RN4220 as a reference (Table 2). One SNP at amino acid position 498 (GCG → GCT) was common to all 16 isolates, which belonged to four different *S. aureus *clonal complexes (CCs) (Table 2). This SNP has also been reported in ST247-MRSA-I control strains ATCCBAA44 and PER88 (CC8), and in ST228-MRSA-I (CC5) isolates from Spain [15]. Codon usage tables derived from genome sequences of six *S. aureus *control strains (NCTC8325, COL, Newman, USA300, N315 and Mu50), indicated that the codon GCT is twice as prevalent as GCG [20]. It is possible that the SNP arose on separate occasions in multiple *S. aureus *lineages and that its prevalence is related to codon bias in this organism. However, it seems more likely that RN4220 contains the SNP (GCT → GCG), which arose once in this strain. This can only be confirmed when more *rpoB *sequences of *S. aureus *isolates from a variety of genetic backgrounds become available.

Of greater interest is the only other conserved silent SNP found in the codon for arginine at amino acid position 512 (CGT → CGC) that was observed in all ST612-MRSA-IV isolates (Table 2). This mutation was notable for two reasons: firstly, AT-rich organisms such as *S*. *aureus *more commonly favour AT-rich codons with either adenine or thymine bases, rather than cytosine, at the third position [21,22]; secondly, codon usage tables indicated that CGT is more common than CGC for arginine [20]. Thus, it is possible to suggest that the SNP (CGT → CGC) has not arisen on multiple occasions in ST612-MRSA-IV, but instead was inherited from a common ancestor and has been conserved within the lineage.

Interestingly, ST612-MRSA-IV has also recently been reported as the predominant clone in a population of horses in Australia [23]. All of the equine ST612-MRSA-IV isolates that were tested were rifampicin-resistant, making it tempting to speculate that they may be related to those described in this study; however, the equine strains carried SCC*mec *type IVa [23], while the ST612-MRSA-IV isolates from Cape Town and Australia carried SCC*mec *type lished data), which suggests at least two separate SCC*mec *acquisitions in this genetic background.

Although mutations associated with resistance frequently evince an initial fitness cost to the organism, it has been shown that rifampicin-resistant *E. coli *do not revert to wild-type susceptibility in the absence of this antibiotic. Rather, they persist because of their capacity to develop compensatory mutations, which restore bacterial fitness [24]. Other studies have also suggested that the reduction of antibiotic pressure may not necessarily result in reversion to susceptibility [25], which is worrying in our setting given that ST612-MRSA-IV is multidrug-resistant [5].

Vancomycin remains the drug of choice for the treatment of multidrug-resistant MRSA infections; however, the emergence of vancomycin-resistant *S. aureus *poses a new challenge. Watanabe *et al. *[17] have suggested that certain mutational changes in *rpoB*, including H_481_Y, may be linked to reduced vancomycin susceptibility in *S. aureus*. In light of these facts, the vancomycin MICs of isolates selected for *rpoB *genotyping in the current study were determined by E-test. Interestingly, the ST5-MRSA-I isolate, with *rpoB *genotype H_481_Y, was susceptible to vancomycin (MIC of 2 mg/L). Of interest is the observation that isolates with MICs of 2 mg/L have been associated with a poor clinical response to vancomycin [26]. All ST612-MRSA-IV were susceptible to vancomycin (MICs of ≤ 1 mg/L), suggesting that the mutational changes present in *rpoB *in these isolates are not associated with resistance to vancomycin.

## Conclusions

A subset of ST612-MRSA-IV isolates from Cape Town hospitals, broadly representative of the total collection with respect to molecular characteristics, as well as the hospital of isolation, was selected to determine the mechanism of rifampicin resistance in this clone. Collectively, the data support a hypothesis of clonal expansion of a rifampicin-resistant ST612-MRSA-IV strain in local hospitals. The data also suggest that these isolates may be related to rifampicin-resistant ST612-MRSA-IV previously described in South Africa and Australia. Studies including additional ST612-MRSA-IV isolates collected from South Africa, Australia and the United Kingdom are required to investigate further the evolution of this clone.

## Authors' contributions

MJJvR and BGE conceived and designed the study. MJJvR carried out the molecular studies. AW co-ordinated clinical aspects of the study. AW also obtained, analysed and interpreted the clinical data. MJJvR and BGE wrote the manuscript, which was critically reviewed by AW. All authors read and approved the final manuscript.
